# Initial Tumor Necrosis Factor-Alpha and Endothelial Activation Are Associated with Hemorrhagic Complications during Extracorporeal Membrane Oxygenation

**DOI:** 10.3390/jcm12134520

**Published:** 2023-07-06

**Authors:** Jin Ho Jang, Kyung-Hwa Shin, Hye Rin Lee, Eunjeong Son, Seung Eun Lee, Hee Yun Seol, Seong Hoon Yoon, Taehwa Kim, Woo Hyun Cho, Doosoo Jeon, Yun Seong Kim, Hye Ju Yeo

**Affiliations:** 1 Division of Pulmonary, Allergy and Critical Care Medicine, Department of Internal Medicine, Pusan National University Yangsan Hospital, Yangsan 50612, Republic of Korea; jjhteen1@naver.com (J.H.J.); vita4250@naver.com (E.S.); crisislee@hanmail.net (S.E.L.); seolhy@hotmail.com (H.Y.S.); drysh79@gmail.com (S.H.Y.); taehwagongju@naver.com (T.K.); popeyes0212@hanmail.net (W.H.C.); sooli10@hanmail.net (D.J.); yskim@pusan.ac.kr (Y.S.K.); 2 Transplantation Research Center and Research Institute for Convergence of Biomedical Science and Technology, Pusan National University Yangsan Hospital, Yangsan 50612, Republic of Korea; hrlee01070@gmail.com; 3 Department of Laboratory Medicine, Pusan National University School of Medicine, Pusan National University Hospital, Busan 49241, Republic of Korea; skyoungh@pusan.ac.kr; 4 Department of Internal Medicine, Pusan National University School of Medicine, Yangsan 50612, Republic of Korea

**Keywords:** extracorporeal membrane oxygenation, hemorrhage, tumor necrosis factor-alpha, endothelium, biomarkers

## Abstract

Studies on inflammatory markers, endothelial activation, and bleeding during extracorporeal membrane oxygenation (ECMO) are lacking. Blood samples were prospectively collected after ECMO initiation from 150 adult patients who underwent ECMO for respiratory failure between 2018 and 2021. After excluding patients who died early (within 48 h), 132 patients were finally included. Their tumor necrosis factor-alpha (TNF-α), tissue factor (TF), soluble thrombomodulin (sTM), and E-selectin levels were measured. A Cox proportional hazards regression model was used to estimate the hazard ratio for hemorrhagic complications during ECMO. The 132 patients were divided into hemorrhagic (*n* = 23, H group) and non-complication (*n* = 109, N group) groups. The sequential organ failure assessment score, hemoglobin level, and ECMO type were included as covariates in all Cox models to exclude the effects of clinical factors. After adjusting for these factors, initial TNF-α, TF, sTM, E-selectin, and activated protein C levels were significantly associated with hemorrhagic complications (all *p* < 0.001). TNF-α, TF, and E-selectin better predicted hemorrhagic complications than the model that included only the aforementioned clinical factors (clinical factors only (area under the curve [AUC]: 0.804), reference; TNF-α (AUC: 0.914); TF (AUC: 0.915); E-selectin (AUC: 0.869)). Conclusions: TNF-α levels were significantly predictive of hemorrhagic complications during ECMO.

## 1. Introduction

Extracorporeal membrane oxygenation (ECMO) is a life-saving therapy used in the critical care of patients with severe heart and respiratory failure [1,2,3]. Although technological advancements have reduced ECMO-related complications, hemorrhagic complications remain challenging [4,5]. The interaction between the hemostatic system and ECMO components involves complex mechanisms [6,7,8]. Despite research efforts to identify biomarkers for predicting hemorrhagic complications, accurate risk assessment remains challenging [9,10,11]. No biomarker has been established as a reliable predictor of hemostatic complications.

ECMO initiation may elicit immediate and complex inflammatory responses. This response involves the activation of the endothelium, which is the inner lining of blood vessels, and the release of proinflammatory cytokines. Consequently, the normal functioning of the coagulation cascade, which is responsible for blood clotting, can be disrupted [8,12,13,14,15]. The interaction between inflammation and coagulation dysfunction has been well-recognized in critically ill patients, including those with sepsis [16]. However, the specific interactions between inflammation and coagulation in patients undergoing ECMO, and their clinical significance, have not been fully elucidated.

Tumor necrosis factor-alpha (TNF-α) is a well-known representative proinflammatory cytokine that can contribute to bleeding by affecting the coagulation cascade and inducing endothelial activation [17,18,19,20,21,22,23,24]. Thus, this can increase the propensity for bleeding events during ECMO. Understanding the effect of initial TNF-α levels after ECMO initiation on the development of bleeding in patients undergoing ECMO is crucial for effective patient management. This study aimed to investigate whether TNF-α levels after ECMO initiation can predict the occurrence of hemorrhagic complications during ECMO and explore their relationship with endothelial activation.

## 2. Materials and Methods

### 2.1. Subjects and Study Design

**Figure 1:**
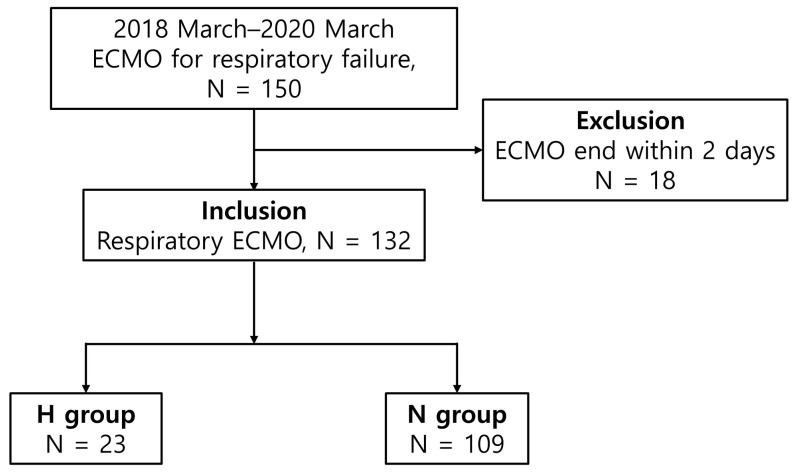
Flowchart of the patient enrollment process.

From March 2018 to March 2021, blood samples were prospectively collected on day one after ECMO initiation and on day 7 after ECMO from 150 adult patients who underwent ECMO for respiratory failure at the Pusan National University Yangsan Hospital Biobank; all patients or their legal guardians provided informed consent for blood collection. The levels of serum TNF-α, other proinflammatory cytokines, and endothelial activation markers were measured using Biobank samples. The clinical data were retrospectively collected and analyzed. There were 150 eligible patients; 18 patients who died within 48 h were excluded, and 132 patients were finally included in the study. The patients were divided into two groups. Patients with hemorrhagic complications during ECMO support were assigned to the H group (*n* = 23), whereas those without hemorrhagic complications during ECMO support were assigned to the N group (*n* = 109) (Figure 1). Hemorrhagic complications were defined as retroperitoneal, pulmonary, or gastrointestinal bleeding, brain or intramuscular hematoma requiring embolization, endoscopic hemostasis, or surgery, and/or a decrease in the hemoglobin level of >2 g/dL over a 24 h period [25]. This study was approved by the Institutional Review Board of Pusan National University Yangsan Hospital (approval no. 05-2022-004).

### 2.2. Sample Collection

Blood samples were collected in serum separator tubes (Greiner Bio-One, Kremsmünster, Austria) or heparin plasma tubes (Greiner Bio-One, Kremsmünster, Austria) and immediately centrifuged at 3000 rpm for 10 min. Aliquots of serum were stored at −80 °C to facilitate batch analysis.

### 2.3. Luminex Assay and Enzyme-Linked Immunosorbent Assay

Human-premixed multianalyte kits (R&D Systems, Minneapolis, MN, USA) were used to determine the levels of the five biomarkers of interest. A kit (catalog number: LXSAHM-06) was used to analyze the coagulation factor III/tissue factor (TF), D-dimer, E-selectin/CD62E, P-selectin/CD62P, and soluble thrombomodulin/BDCA-3 levels. The samples were diluted 2-fold using the dilution buffer provided in the kit. All standards and samples were assayed in duplicates. The antibody-coupled beads were incubated with serum, biotinylated detection antibodies, and streptavidin–phycoerythrin. The samples were then evaluated using a laser-based fluorescent analytical test instrument (Luminex^®^ 100™, Luminex Corp., Austin, TX, USA). Serum levels of interleukin-1β, TNF-α, platelet factor 4, and β-thrombogloblin were determined using enzyme immunoassays per the manufacturer’s instructions (Table S1). Plasma levels of activated protein C (APC) and thrombin–throbomodulin (thrombin-TM) complex were also determined using enzyme immunoassays per the manufacturer’s instructions (Table S1).

### 2.4. Heparin Protocol during ECMO

The ECMO-related protocols are shown in Supplement 2. The ECMO system consisted of a polymethylpentene fiber oxygenator system (Quadrox PLS; Maquet Inc., Hirrlingen, Germany) with simplified bioline-coated circuits (Maquet Inc., Hirrlingen, Germany). All patients were supported by centrifugal pumps (Maquet Inc.). Patients received an initial unfractionated heparin bolus of 50 U/kg body weight when the cannula was placed, and unfractionated heparin was continuously infused during ECMO. Heparin infusion was regulated to maintain an activated partial thromboplastin time (aPTT) of 80–90 s, measured every 6 h. In the event of bleeding, the aPTT target was adjusted to 60–70 s. If the bleeding persisted, anticoagulation therapy was discontinued and restarted after the bleeding stopped. If heparin-induced thrombocytopenia was suspected, a heparin-platelet factor 4 antibody test was performed; unfractionated heparin was discontinued, and argatroban administered to maintain an aPTT of 80–90 s. Moreover, our primary choice for anticoagulation in renal replacement therapy (RRT) was unfractionated heparin. The RRT filter was prepared by priming it with 1 L of heparinized saline containing 5000 U of heparin without an initial loading dose. The ongoing maintenance dose was administered at a rate of 250 U/h (or adjusted to 5 U/kg) with further adjustments based on aPTT measurements. Additionally, RRT was performed without using anticoagulants in patients with bleeding complications. Transfusions were performed when the platelet count was <80,000/μL or the hemoglobin level was <8 g/dL.

### 2.5. Statistical Analysis

All statistical analyses were performed using MedCalc, version 11.3.6.0 (MedCalc Software, Mariakerke, Belgium) and SPSS, version 26 (IBM Corp., Armonk, NY, USA). Categorical variables were presented as numbers with percentages, and continuous variables were presented as means and standard deviations or medians and interquartile ranges, as appropriate. Continuous variables were compared using Student’s *t*-test (parametric values) or the Mann–Whitney U test. Categorical variables were compared using the χ^2^ test or Fisher’s exact test, as appropriate. A Cox proportional hazards regression model was used to estimate the hazard ratios (HRs) and 95% confidence intervals (CIs) for the development of hemorrhagic complications during ECMO. All continuous variables were standardized to zero mean and unit variance to ensure a standard scale. The sequential organ failure assessment (SOFA) score before ECMO initiation, ECMO type, and hemoglobin level were included as covariates in all Cox models. Receiver operating characteristic (ROC) curve analysis was used to evaluate the predictive performances of the Cox regression models. The clinical-factor-only Cox model included the following factors: SOFA score before ECMO initiation, ECMO type, and hemoglobin level. A one-sided DeLong’s test [26] was conducted to compare the difference in the area under the ROC curve (AUC) between the Cox models using TNF-α and endothelial markers and the clinical-factor-only model. A one-sided test was performed because the AUC of the Cox model, including TNF-α and endothelial markers, was expected to be greater than that of the clinical-factor-only Cox model. Pearson’s correlations were calculated to evaluate the correlations between TNF-α, TF, soluble thrombomodulin (sTM), thrombin-TM complex, and APC. All tests were two-tailed, and the level of statistical significance was set at *p* < 0.05.

## 3. Results

### 3.1. Baseline Clinical Characteristics according to Hemorrhagic Complications

Of the 132 patients, 23 developed hemorrhagic complications. Pulmonary hemorrhage was the most common (5.3%), followed by retroperitoneal (3.8%), intramuscular (3%), gastrointestinal (3%), brain (1.5%), and cannula (0.8%) hemorrhages. The onset of hemorrhagic complications occurred after a median of 11 days (interquartile range: 6–15 days) of ECMO. The baseline clinical characteristics of patients stratified according to hemorrhagic complications are presented in Table 1. Among the 132 patients, 112 (84.8%) received venovenous ECMO, 17 (12.9%) had venoarterial ECMO, and 3 (2.3%) received venoarterial venous ECMO. There was no significant difference in ECMO modalities between the H and N groups (*p* = 0.580). However, the SOFA score before ECMO initiation was significantly higher in the H group than in the N group (11.5 ± 1.9 vs. 10.4 ± 3.0, *p* = 0.028). The Acute Physiology and Chronic Health Evaluation II score, Charlson Comorbidity Index, and the baseline laboratory results, except for D-dimer levels, did not differ significantly between the two groups. Baseline D-dimer concentration was significantly higher in the H group than in the N group (mean fluorescence intensity: 7906 vs. 6900, *p* = 0.005). The anticoagulation method used did not differ significantly between the two groups. RRT was performed significantly more frequently in the H group than in the N group (52.2% vs. 28.4%; *p* = 0.027). The mean duration of ECMO was longer in the H group than in the N group (26.0 ± 13.9 days vs. 14.6 ± 11.4 days, *p* < 0.001). There were no differences in heparin dose, antithrombin III activity, and platelet count between the two groups on D1 and D7 (Figure S2).

### 3.2. Clinical Outcomes according to Hemorrhagic Complications

There was no significant difference in the proportion of bridge to transplantation or recovery between the two groups (Table S2). The differences in intensive care unit stay and hospital mortality rates were also not significant. During ECMO, the transfusion volumes of red blood cells, fresh frozen plasma, and platelets were significantly higher in the H group than in the N group (red blood cells: 7 units vs. 2 units, *p* < 0.001; fresh frozen plasma: 3 units vs. 1 unit, *p* < 0.001; platelets: 4 units vs. 1 unit, *p* < 0.001). There was no significant difference in the incidence of major thrombotic complications between the two groups (26.1% vs. 17.4%, *p* = 0.381).

### 3.3. Days 1 and 7 Inflammation, Endothelial, and Platelet Activation Markers

**Figure 2:**
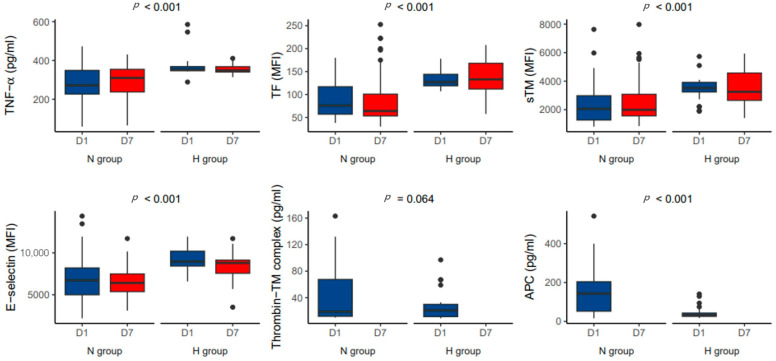
Comparison of the levels of TNF-α and endothelial activation markers according to hemorrhagic complications on day 1 and 7 after initiation of extracorporeal membrane oxygenation.

On day 1 after ECMO initiation, TNF-α, TF, sTM, and E-selectin levels were significantly higher in the H group than in the N group (*p* < 0.001; Figure 2). The differences in P-selectin, interleukin-1β, platelet factor 4, and β-thromboglobulin levels between the two groups were not statistically significant (Figure S2). Similarly, on day 7, the mean TNF-α, TF, sTM, E-selectin, and P-selectin levels were significantly higher in the H group than in the N group (*p* < 0.001; Figure 2).

### 3.4. Relationship between TNF-α and Thrombomodulin/APC System after ECMO Initiation

**Figure 3:**
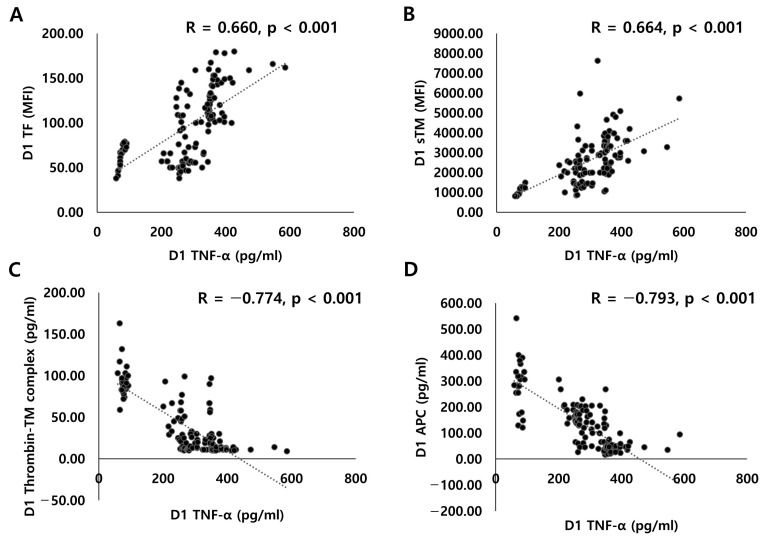
Correlations between TNF-α and endothelial activation markers: (**A**) plasma TNF-α and TF levels; (**B**) plasma TNF-α and sTM levels; (**C**) plasma TNF-α and thrombin-TM complex levels; (**D**) plasma TNF-α and APC levels.

The APC level was significantly lower in the H group than in the N group (45.3 vs. 153.7 pg/mL, *p* < 0.001; Figure 2). The TNF-α level was positively correlated with the TF (r = 0.660, *p* < 0.001, Figure 3A) and sTM (r = 0.664, *p* < 0.001; Figure 3B) levels. The TNF-α level was negatively correlated with the thrombin-TM complex (r = −0.774, *p* < 0.001; Figure 3C) and APC (r = −0.793, *p* < 0.001; Figure 3D) levels.

### 3.5. Associations of Initial TNF-α, Endothelial Markers, and Hemorrhagic Complications during ECMO

**Figure 4:**
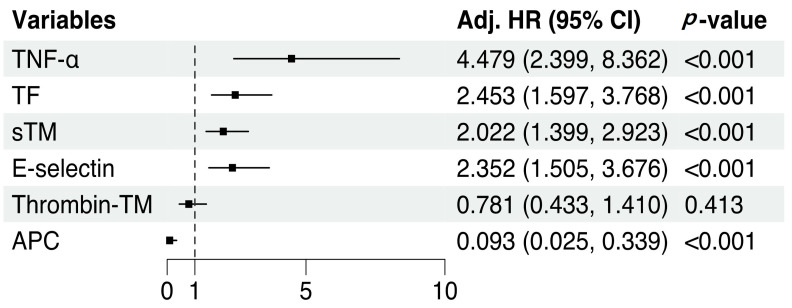
Cox regression analysis adjusted for clinical factors. All variables were standardized to zero mean and unit variance to achieve a standard scale. SOFA score before ECMO initiation, hemoglobin level, and ECMO type were included as covariates in all Cox models.

To exclude the effects of clinical factors, SOFA score, hemoglobin, and ECMO types were included as covariates in all Cox models. After adjustment for clinical factors, initial TNF-α (adjusted HR: 4.48, 95% CI: 2.40–8.36, *p* < 0.001), TF (adjusted HR: 2.45, 95% CI: 1.60–3.77, *p* < 0.001), sTM (adjusted HR: 2.02, 95% CI: 1.40–2.92, *p* < 0.001), E-selectin (adjusted HR: 2.35, 95% CI: 1.51–3.68, *p* < 0.001), and APC (adjusted HR: 0.09, 95% CI: 0.03–0.34, *p* < 0.001) levels were significantly associated with hemorrhagic complications during ECMO (Figure 4).

### 3.6. Predictive Performances of TNF-α and Endothelial Markers for Hemorrhagic Complications during ECMO

**Figure 5:**
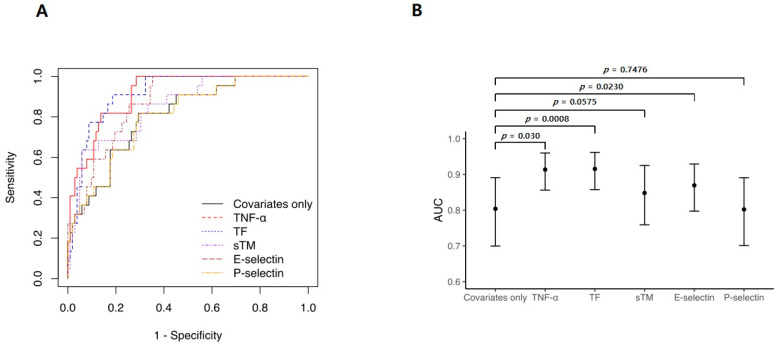
Adjusted receiver operating characteristic (ROC) curves in Cox regression analyses. (**A**) ROC curve analysis was used to evaluate the prediction performance of the Cox regression models. (**B**) Compared with the model using only clinical factors, adding TF or TNF-α improved the area under the curve by ~11%. Clinical-factor-only model refers to a Cox model that includes only the following factors: sequential organ failure assessment score before ECMO initiation, hemoglobin level, and ECMO type.

ROC curve analysis was performed to evaluate the predictive performance of the Cox regression models (Figure 5A). Significantly higher AUC values were observed for the models that included TNF-α, TF, and E-selectin levels than for the clinical-factor-only model (clinical-factor-only (AUC: 0.804, 95% CI: 0.700–0.891), reference; TNF-α (AUC: 0.914, 95% CI: 0.856–0.960, *p* = 0.003); TF (AUC: 0.915, 95% CI: 0.857–0.962, *p* = 0.001); E-selectin (AUC: 0.869, 95% CI: 0.797–0.929, *p* = 0.023), Figure 5B).

## 4. Discussion

This study evaluated the ability of proinflammatory cytokines and endothelial activation on day 1 after ECMO initiation to predict the development of hemorrhagic complications during ECMO. The levels of TNF-α and endothelial markers on day 1 were significantly correlated, indicating that coagulation cascades were activated. After adjusting for critical clinical factors, TNF-α and endothelial activation markers remained associated with hemorrhagic complications during ECMO. The levels of these markers predicted the development of hemorrhagic complications during ECMO with a predictive power better than that of the model that consisted of only clinical factors.

Patients experiencing acute respiratory failure often display hyperinflammation and endothelial activation in the lungs and other organs [27]. This dysregulation of the inflammatory response and endothelial function can significantly affect the coagulation cascade, potentially resulting in an elevated risk of bleeding [28]. Moreover, extracorporeal circulation, such as ECMO, can significantly affect these responses by interacting with the patient’s blood and artificial circuit [8,29,30]. So far, there is a lack of studies explicitly examining proinflammatory cytokines and endothelial markers in patients undergoing respiratory ECMO. A recent study observed that elevated levels of TNF-α in patients with left ventricular assist devices were associated with TF expression and nonsurgical bleeding [31]. Consistent with these observations, our findings support the notion that high levels of TNF-α after ECMO initiation may be associated with an imbalance in the endothelial-activated coagulation system. This imbalance can disrupt the delicate equilibrium of the coagulation cascade, leading to a heightened risk of bleeding during ECMO support [21].

Several biomarkers have been proposed to predict hemorrhagic complications during ECMO [32,33]. In this study, the performance of fibrinogen (AUC: 0.79, *p* = 0.480) and D-dimer (AUC: 0.64, *p* = 0.062) as biomarkers did not align with previous findings (Figure S2). This discrepancy may be related to the differences in patient characteristics, underlying diseases, and inflammatory states. In contrast to the present study, previous studies mainly included patients with myocarditis or cardiac arrest supported by venoarterial ECMO [32,33], which mainly consisted of patients with ILD (51%) and high levels of inflammation, such as acute respiratory distress syndrome and pneumonia. In this study, we aimed to evaluate the clinical significance of TNF-α as a potential biomarker, for predicting hemorrhagic complications in patients undergoing ECMO [34,35]. Our results revealed that hemorrhagic complications were independently related to TNF-α and markers of endothelial activation (Figure 4). The ability to predict hemorrhagic complications improved by approximately 11% when TNF-α or TF levels were entered into a model that consisted only of clinical factors (Figure 5B). Thus, TNF-α and TF levels may be biomarkers for identifying patients at high risk of hemorrhagic complications. These findings will help refine risk stratification strategies, optimize patient management, and guide decision making regarding anticoagulation therapy and bleeding prevention in patients undergoing ECMO. A multicenter prospective study with a larger cohort is required to validate our results in all patients undergoing ECMO, including those with cardiac failure.

This study has several limitations. First, because this was a single-center retrospective study, the possibility of selection bias cannot be ruled out. Additionally, half of the cohort had ILD, and all patients were Asian; thus, our findings may not be representative of all patients on ECMO. Second, measurements of proinflammatory and endothelial markers on day 1 did not reflect the dynamic interactions between inflammation and coagulation during ECMO support. Despite those limitations, the important clinical factors were well-calibrated in our study, such that we could convincingly demonstrate the independent performances of TNF-α and endothelial activation markers. The advantages of our study include the inclusion of a study population with severe organ dysfunction, the presence of various etiologies of respiratory failure, and a larger number of patients than in previous studies. To the best of our knowledge, this study is the first to show that inflammation-induced changes in the hemostatic system that occur after ECMO initiation can affect the onset of coagulation-related hemorrhagic complications during ECMO. Thus, the novel biomarkers identified in this study may serve as therapeutic targets for preventing hemorrhagic complications. However, further in vivo studies are needed to determine whether the inhibition of endothelial activation can reduce hemorrhagic complications during ECMO.

## 5. Conclusions

This study provides valuable insights into the clinical significance of initial inflammation and endothelial activation in developing hemorrhagic complications during ECMO. Specifically, the interaction between TNF-α and endothelial dysfunction may be crucial in this process. By assessing the TNF-α level after the initiation of ECMO, clinicians may be able to identify patients more susceptible to bleeding events and implement appropriate management strategies. Further research is needed to explore longitudinal changes in these biomarkers during ECMO and their correlation with hemorrhagic complications. By obtaining a more detailed and dynamic profile of inflammation, endothelial markers, and coagulation activity, clinicians can tailor interventions and optimize patient care during ECMO.

## Figures and Tables

**Table 1:** Baseline characteristics, stratified according to hemorrhagic complications during extracorporeal membrane oxygenation.

Variables	Total (*n* = 132)	H Group (*n* = 23)	N Group (*n* = 109)	*p*-Value
Age, years	57 (50–63)	54 (51–66)	57 (49–62)	0.759
Male sex	77 (58.3%)	12 (52.2%)	65 (59.6%)	0.510
BMI, kg/m^2^	22.7 ± 4.5	23.6 ± 4.6	22.5 ± 4.5	0.295
Diagnosis				0.386
Viral pneumonia	11 (8.3%)	2 (8.7%)	9 (8.3%)	
Bacterial pneumonia	26 (19.7%)	2 (8.7%)	24 (22.0%)	
Asthma	1 (0.8%)	0 (0.0%)	1 (0.9%)	
ILD	68 (51.5%)	11 (47.8%)	57 (52.3%)	
Sepsis	20 (15.2%)	6 (26.1%)	14 (12.8%)	
Others ^a^	6 (4.5%)	2 (8.7%)	4 (3.7%)	
ECMO modalities				0.580
VV	112 (84.8%)	19 (82.6%)	93 (85.3%)	
VA	17 (12.9%)	4 (17.4%)	13 (11.9%)	
VAV	3 (2.3%)	0 (0.0%)	3 (2.8%)	
Mean blood flow on 1st day of ECMO (L/min)	3.1 ± 0.9	3.0 ± 1.4	3.2 ± 0.7	0.548
Mean sweep gas on 1st day of ECMO (L/min)	4.2 ± 1.4	4.1 ± 1.6	4.2 ± 1.4	0.926
SOFA score before ECMO initiation	10.6 ± 2.8	11.5 ± 1.9	10.4 ± 3.0	0.028
APACHE II	13.4 ± 6.4	13.0 ± 3.6	13.5 ± 6.9	0.590
CCI	1.7 ± 1.5	1.8 ± 1.1	1.7 ± 1.6	0.828
Baseline laboratory findings				
Plasma Hb (mg/dL)	16.3 ± 13.4	14.9 ± 8.3	16.5 ± 14.0	0.703
WBC (×10^3^/μL)	12.1 ± 7.4	12.7 ± 8.1	12.0 ± 7.3	0.664
PLT (×10^3^/mm^3^)	173.6 ± 91.4	186.3 ± 122.5	171.0 ± 84.2	0.478
CRP (mg/dL)	12.8 ± 9.5	13.1 ± 10.8	12.7 ± 9.44	0.896
D-dimer (ng/mL)	6986.6 (5539–7874.6)	7906 (6967–7906)	6900 (5539–7787)	0.005
Fibrinogen (mg/dL)	412.1 ± 134.9	383.0 ± 158.0	417.4 ± 130.5	0.336
PT/INR	1.3 ± 0.3	1.3 ± 0.1	1.3 ± 0.4	0.972
aPTT (s)	74.9 ± 33.0	73.6 ± 40.5	75.2 ± 31.5	0.846
Anticoagulation				0.316
Heparin	117 (88.6%)	19 (82.6%)	98 (89.9%)	
Argatroban	15 (11.4%)	4 (17.4%)	11 (10.1%)	
RRT	43 (32.6%)	12 (52.2%)	31 (28.4%)	0.027
ECMO duration, days	16.6 ± 12.6	26.0 ± 13.9	14.6 ± 11.4	<0.001
Long-term ECMO ^b^	67 (50.8%)	18 (78.3%)	49 (45.0%)	0.004

Data are presented as mean ± standard deviation or number (%). BMI: body mass index, ILD: interstitial lung disease, ECMO: extracorporeal membrane oxygenation, VV: venovenous, VA: venoarterial, VAV: venoarterial venous, SOFA: sequential organ failure assessment, APACHE II: Acute Physiology and Chronic Health Evaluation II, CCI: Charlson Comorbidity Index, WBC: white blood cells, PLT: platelets, CRP: C-reactive protein, PT/INR: prothrombin international normalized ratio, aPTT: activated partial thromboplastin time, Hb: hemoglobin, RRT: renal replacement therapy. ^a^ Pulmonary tuberculosis, bronchiolitis obliterans syndrome, primary pulmonary hypertension, Pneumocystis pneumonia, and pulmonary thromboembolism. ^b^ Use of ECMO for > 2 weeks.

## Data Availability

The datasets used and/or analyzed in the current study are available from the corresponding author upon reasonable request.

## Supplementary Materials

The following supporting information can be downloaded at: https://www.mdpi.com/article/10.3390/jcm12134520/s1, Table S1: ELISA Kit; Supplement 2: ECMO-related protocols. Refs. [36,37] are cited in the supplemental file; Table S2: Clinical outcomes according to the hemorrhagic complications; Figure S1: Heparin dose, antithrombin III activity, and platelet count between the two groups; Figure S2: Comparison of Inflammation markers, endothelial markers and platelet activation markers at D1 on ECMO; Figure S3: ROC curve analysis of inflammatory markers and platelet activation markers predicting hemorrhagic complications during ECMO.

## References

[1] Brodie D. (2018). The Evolution of Extracorporeal Membrane Oxygenation for Adult Respiratory Failure. Ann. Am. Thorac. Soc..

[2] Yeo H.J., Jeon D., Kim Y.S., Cho W.H., Kim D. (2016). Veno–veno–arterial extracorporeal membrane oxygenation treatment in patients with severe acute respiratory distress syndrome and septic shock. Crit. Care.

[3] Rajsic S., Treml B., Jadzic D., Breitkopf R., Oberleitner C., Krneta M.P., Bukumiric Z. (2022). Extracorporeal membrane oxygenation for cardiogenic shock: A meta-analysis of mortality and complications. Ann. Intensiv. Care.

[4] Thomas J., Kostousov V., Teruya J. (2018). Bleeding and Thrombotic Complications in the Use of Extracorporeal Membrane Oxygenation. Semin. Thromb. Hemost..

[5] Villalba C.A.F., McMullan D.M., Reed R.C., Chandler W.L. (2022). Thrombosis in Extracorporeal Membrane Oxygenation (ECMO) Circuits. ASAIO J..

[6] Doyle A.J., Hunt B.J. (2018). Current Understanding of How Extracorporeal Membrane Oxygenators Activate Haemostasis and Other Blood Components. Front. Med..

[7] Murphy D.A., Hockings L.E., Andrews R.K., Aubron C., Gardiner E.E., Pellegrino V.A., Davis A.K. (2015). Extracorporeal Membrane Oxygenation—Hemostatic Complications. Transfus. Med. Rev..

[8] Millar J.E., Fanning J.P., McDonald C.I., McAuley D.F., Fraser J.F. (2016). The inflammatory response to extracorporeal membrane oxygenation (ECMO): A review of the pathophysiology. Crit. Care.

[9] Panigada M., Meli A., Scotti E., Properzi P., Brioni M., Kamel S., Ghirardello S., Scudeller L., Dalton H.J., Grasselli G. (2021). Viscoelastic Coagulation Monitor as a Novel Device to Assess Coagulation at the Bedside. A Single-Center Experience During the COVID-19 Pandemic. ASAIO J..

[10] Chandel A., Patolia S., Looby M., Bade N., Khangoora V., King C.S. (2021). Association of D-dimer and Fibrinogen With Hypercoagulability in COVID-19 Requiring Extracorporeal Membrane Oxygenation. J. Intensiv. Care Med..

[11] Dornia C., Philipp A., Bauer S., Stroszczynski C., Schreyer A.G., Müller T., Koehl G.E., Lehle K. (2015). D-dimers Are a Predictor of Clot Volume Inside Membrane Oxygenators During Extracorporeal Membrane Oxygenation. Artif. Organs.

[12] Graulich J., Walzog B., Marcinkowski M., Bauer K., Kössel H., Fuhrmann G., Bührer C., Gaehtgens P., Versmold H.T. (2000). Leukocyte and Endothelial Activation in a Laboratory Model of Extracorporeal Membrane Oxygenation (ECMO). Pediatr. Res..

[13] Zhang M., Pauls J.P., Bartnikowski N., Haymet A.B., Chan C.H.H., Suen J.Y., Schneider B., Ki K.K., Whittaker A.K., Dargusch M.S. (2021). Anti-thrombogenic Surface Coatings for Extracorporeal Membrane Oxygenation: A Narrative Review. ACS Biomater. Sci. Eng..

[14] Ki K.K., Millar J.E., Langguth D., Passmore M.R., McDonald C.I., Shekar K., Shankar-Hari M., Cho H.J., Suen J.Y., Fraser J.F. (2020). Current Understanding of Leukocyte Phenotypic and Functional Modulation During Extracorporeal Membrane Oxygenation: A Narrative Review. Front. Immunol..

[15] Al-Fares A., Pettenuzzo T., Del Sorbo L. (2019). Extracorporeal life support and systemic inflammation. Intensiv. Care Med. Exp..

[16] Dellinger R.P. (2003). Inflammation and Coagulation: Implications for the Septic Patient. Clin. Infect. Dis..

[17] Salat C., Boekstegers P., Holler E., Werdan K., Reinhardt B.S., Fateh-Moghadam S., Pihusch R., Kaul M., Beinert T., Hiller E. (1996). Hemostatic Parameters in Sepsis Patients Treated with Anti-TNF±-Monoclonal Antibodies. Shock.

[18] Gao X., Belmadani S., Picchi A., Xu X., Potter B.J., Tewari-Singh N., Capobianco S., Chilian W.M., Zhang C. (2007). Tumor Necrosis Factor-α Induces Endothelial Dysfunction in Lepr(db) Mice. Circulation.

[19] Kirchhofer D., Tschopp T.B., Hadváry P., Baumgartner H.R. (1994). Endothelial cells stimulated with tumor necrosis factor-alpha express varying amounts of tissue factor resulting in inhomogenous fibrin deposition in a native blood flow system. Effects of thrombin inhibitors. J. Clin. Investig..

[20] Lentz S.R., Tsiang M., Sadler J.E. (1991). Regulation of thrombomodulin by tumor necrosis factor-alpha: Comparison of tran-scriptional and posttranscriptional mechanisms. Blood.

[21] Ikezoe T. (2015). Thrombomodulin/activated protein C system in septic disseminated intravascular coagulation. J. Intensiv. Care.

[22] Nan B., Lin P., Lumsden A.B., Yao Q., Chen C. (2005). Effects of TNF-α and curcumin on the expression of thrombomodulin and endothelial protein C receptor in human endothelial cells. Thromb. Res..

[23] Sohn R.H., Deming C.B., Johns D.C., Champion H.C., Bian C., Gardner K., Rade J.J. (2005). Regulation of endothelial thrombomodulin expression by inflammatory cytokines is mediated by activation of nuclear factor-kappa B. Blood.

[24] Conway E.M., Rosenberg R.D. (1988). Tumor necrosis factor suppresses transcription of the thrombomodulin gene in endothelial cells. Mol. Cell Biol..

[25] Karagiannidis C., Brodie D., Strassmann S., Stoelben E., Philipp A., Bein T., Müller T., Windisch W. (2016). Extracorporeal membrane oxygenation: Evolving epidemiology and mortality. Intensiv. Care Med..

[26] Delong E.R., Delong D.M., Clarke-Pearson D.L. (1988). Comparing the Areas under Two or More Correlated Receiver Operating Characteristic Curves: A Nonparametric Approach. Biometrics.

[27] Frantzeskaki F., Armaganidis A., Orfanos S.E. (2017). Immunothrombosis in Acute Respiratory Distress Syndrome: Cross Talks between Inflammation and Coagulation. Respiration.

[28] Delabranche X., Helms J., Meziani F. (2017). Immunohaemostasis: A new view on haemostasis during sepsis. Ann. Intensiv. Care.

[29] Vassiliou A.G., Kotanidou A., Dimopoulou I., Orfanos S.E. (2020). Endothelial Damage in Acute Respiratory Distress Syndrome. Int. J. Mol. Sci..

[30] Birnhuber A., Fliesser E., Gorkiewicz G., Zacharias M., Seeliger B., David S., Welte T., Schmidt J., Olschewski H., Wygrecka M. (2021). Between inflammation and thrombosis: Endothelial cells in COVID-19. Eur. Respir. J..

[31] Tabit C., Coplan M., Chen P., Jeevanandam V., Uriel N., Liao J. (2017). Increased Tumor Necrosis Factor-α Levels in Patients with Continuous-Flow Left Ventricular Assist Devices Mediate Vascular Instability and Are Associated with Higher Non-Surgical Bleeding. J. Heart Lung Transplant..

[32] Nguyen T.P., Phan X.T., Nguyen T.H., Huynh D.Q., Tran L.T., Pham H.M., Nguyen T.N., Kieu H.T., Pham T.T.N. (2022). Major Bleeding in Adults Undergoing Peripheral Extracorporeal Membrane Oxygenation (ECMO): Prognosis and Predictors. Crit. Care Res. Pract..

[33] Otani T., Sawano H., Natsukawa T., Matsuoka R., Nakashima T., Takahagi M., Hayashi Y. (2018). D-dimer predicts bleeding complication in out-of-hospital cardiac arrest resuscitated with ECMO. Am. J. Emerg. Med..

[34] Le Guennec L., Cholet C., Huang F., Schmidt M., Bréchot N., Hékimian G., Besset S., Lebreton G., Nieszkowska A., Leprince P. (2018). Ischemic and hemorrhagic brain injury during venoarterial-extracorporeal membrane oxygenation. Ann. Intensiv. Care.

[35] Aubron C., DePuydt J., Belon F., Bailey M., Schmidt M., Sheldrake J., Murphy D., Scheinkestel C., Cooper D.J., Capellier G. (2016). Predictive factors of bleeding events in adults undergoing extracorporeal membrane oxygenation. Ann. Intensiv. Care.

[36] (2017). Extracorporeal Life Support Organization (ELSO) General Guidelines for all ECLS Cases. Version 1.4. http://wwwelsoorg/resources/guidelinesaspx.

[37] Yeo H.J., Cho W.H., Kim D. (2019). Learning curve for multidisciplinary team setup in veno-venous extracorporeal membrane oxygenation for acute respiratory failure. Perfusion.

