# Supplementary material for: Initial Tumor Necrosis Factor-Alpha and Endothelial Activation Are Associated with Hemorrhagic Complications during Extracorporeal Membrane Oxygenation
Source: J Clin Med. 2023 Jul 6;12(13):4520. doi: 10.3390/jcm12134520 (PMC10342556; doi:10.3390/jcm12134520)
Supplement: Supplementary file 1 [file jcm-12-04520-s001.zip › jcm-2415066-supplementary.pdf]

Supplementary materials

Initial tumor necrosis factor-alpha and endothelial activation are associated with hemorrhagic complications during extracorporeal membrane oxygenation

Jin Ho Jang, Kyung-Hwa Shin, Hye Rin Lee, Eun Jeong Son, Seung Eun Lee, Hee Yun Seol, Seong Hoon Yoon, Taehwa Kim, Woo Hyun Cho, Doosoo Jeon, Yun Seong Kim, Hye Ju Yeo

Tables of Contents

Contents	Page
Table S1. ELISA Kit	3
Supplement 2. ECMO-related protocols	
Table S2. Clinical outcomes according to the hemorrhagic complications	4
Figure S1. Heparin dose, antithrombin III activity, and platelet count between the two groups	5
Figure S2. Comparison of Inflammation markers, endothelial markers and platelet activation markers at D1 on ECMO	7
Figure S3. ROC curve analysis of inflammatory markers and platelet activation markers predicting hemorrhagic complications during ECMO	8

Table S1. ELISA Kit

Markers	ELISA kit
**Human IL-1β**	MyBioSource. San Diego, CA, USA, Cat. No: MBS2884829
**Human TNF-α**	MyBioSource. San Diego, CA, USA, Cat. No: MBS2502004
**Human β-TG**	MyBioSource. San Diego, CA, USA, Cat. No: MBS 762705
**human PF4**	MyBioSource. San Diego, CA, USA, Cat. No: MBS 3803480
**human APC**	MyBioSource. San Diego, CA, USA, Cat. No: MBS283174
**human thrombin-TM complex**	MyBioSource. San Diego, CA, USA, Cat. No: MBS047233

IL-1β, interleukin-1β; TNF-α, tumor necrosis factor-α; β-TG, β-thromboglobulin; PF4, platelet factor 4; APC, activated protein C; TM, thrombomodulin.

Supplement 2. ECMO-related protocols

ECMO protocols were implemented in accordance with the Extracorporeal Life Support Organization's guidelines (1). Bedside care for patients undergoing ECMO was delivered by a multidisciplinary intensive care team (2). All catheters were managed with a standardized protocol and inserted peripherally by an experienced cardiothoracic surgeon, using the Seldinger approach under ultrasound guidance.

References

1. Extracorporeal life support organization (ELSO) general guidelines for all ECLS cases. version 1.4. Available at: http://wwwelsoorg/resources/guidelinesaspx. 2017; Accessed April 22, 2022.
2. Yeo HJ, Cho WH, Kim D. Learning curve for multidisciplinary team setup in veno-venous extracorporeal membrane oxygenation for acute respiratory failure. Perfusion. 2019;34(1_suppl):30-8.

Table S2. Clinical outcomes according to the hemorrhagic complications

Variables	Total (n=132)	H group (n=23)	N group (n=109)	P-value
**Bridge to recovery**	52 (39.4%)	6 (26.1%)	46 (42.2%)	0.151
**Bridge to transplantation**	66 (50.0%)	14 (60.9%)	52 (47.7%)	0.251
**ICU mortality**	33 (25.0%)	7 (30.4%)	26 (23.9%)	0.508
**Hospital mortality**	43 (32.6%)	10 (43.5%)	33 (30.3%)	0.220
**Transfusion**				
**RBC**	3 (1-13.8)	7 (2-18)	2 (1-11.5)	<0.001
**FFP**	1 (0-7.8)	3 (1-10)	1 (0-6.5)	<0.001
**PLT**	1.25 (0-6)	4 (1-7)	1 (0-6)	<0.001
**Major thrombotic complication**[a]	25 (18.9%)	6 (26.1%)	19 (17.4%)	0.381†
**DIC**	23 (17.4%)	4 (17.4%)	19 (17.4%)	1.000

Data are presented as N (%) or median (interquartile range).

†Fisher's exact test was conducted.

ICU, intensive care unit; RBC, red blood cell; FFP, fresh frozen plasma; PLT, platelet; DIC, disseminated intravascular coagulation.

[a]Major thrombotic complications were defined as thrombotic complications in major organs, including cerebral infarction, spinal infarction, pulmonary thromboembolism, deep vein thrombosis, splenic infarction, and arterial thrombosis.

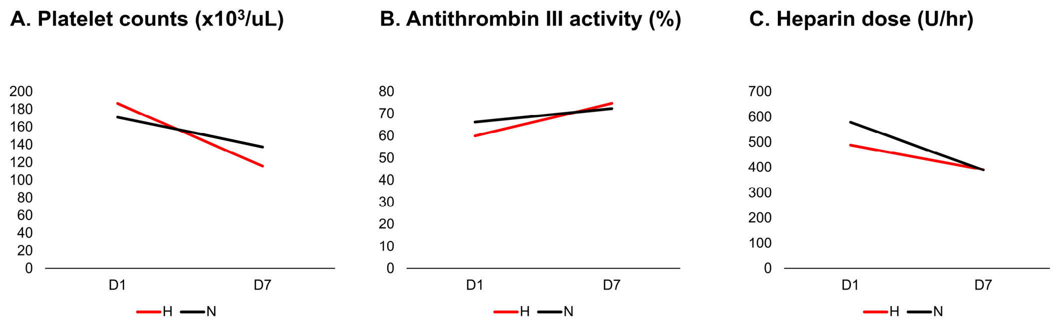

Figure S1. Heparin dose, antithrombin III activity, and platelet count between the two groups

There were no differences in heparin dose, antithrombin III activity, and platelet count between the two groups on D1 and D7 (Figure S2).

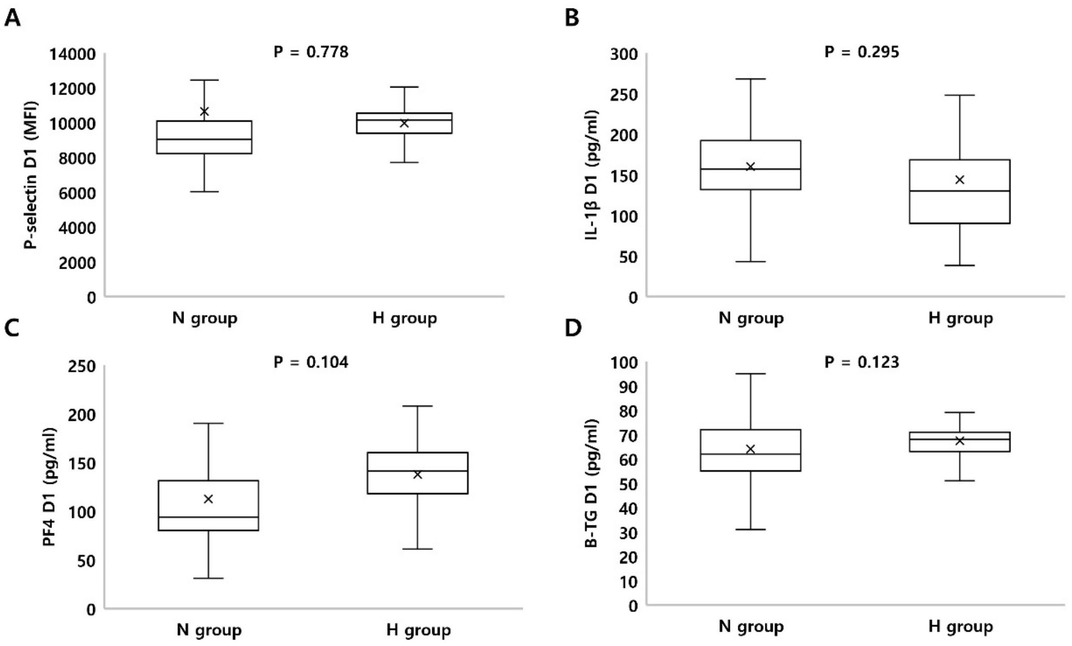

Figure S2. Comparison of inflammation markers, endothelial markers and platelet activation markers at D1 on ECMO. There were no significant differences of P-selectin, IL-1β, PF4 and β-TG between two groups.

IL-1β, interleukin-1β; PF4, platelet factor 4; β-TG, β-thromboglobulin.

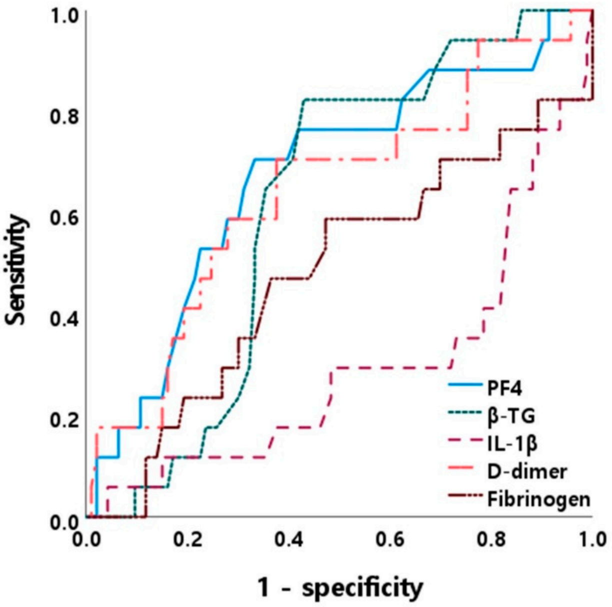

	AUC	P
**PF4**	0.607	0.023
**β-TG**	0.615	0.133
**IL-1β**	0.293	0.007
**D-dimer**	0.643	0.062
**Fibrinogen**	0.794	0.480

Figure S3. ROC curve analysis of inflammatory markers and platelet activation markers predicting hemorrhagic complications during ECMO. PF4 and D-dimer showed significantly poor discriminated hemorrhagic complications.

PF4, platelet factor 4; β-TG, β-thromboglobulin; IL-1β, interleukin-1β.
